# Burden and outcome of respiratory morbidities among children and adolescents with sickle cell disease—A retrospective review of emergency presentations in some Nigerian tertiary institutions

**DOI:** 10.1371/journal.pone.0303323

**Published:** 2024-05-16

**Authors:** Rasheedat Mobolaji Ibraheem, Mohammed Baba Abdulkadir, Rasaki Aliu, Amudalat Issa, Olayinka Rashid Ibrahim, Abdulafeez Oyesola Bello, Fatima Ishaq Abubakar, Iso Precious Oloyede, Yetunde Toyin Olasinde, Datonye Christopher Briggs, Muhammad Faruk Bashir, Qasim Olakunle Salau, Bilkisu Ilah Garba, Hafsat Abolore Ameen, Mohammed Bello Suleiman, Temitayo Olubunmi Bewaji, Hassan Kamiludeen Shina

**Affiliations:** 1 Department of Paediatrics & Child Health, University of Ilorin and University of Ilorin Teaching Hospital, Ilorin, Kwara State, Nigeria; 2 Department of Paediatrics, Gombe State University & Federal Teaching Hospital Gombe, Gombe, Gombe State, Nigeria; 3 Department of Paediatrics, Children Specialist Hospital Centre-igboro, Ilorin, Kwara State, Nigeria; 4 Department of Paediatrics, Federal Medical Centre Katsina, Katsina, Katsina State, Nigeria; 5 Department of Paediatrics, Federal Medical Centre Bida, Bida, Niger State, Nigeria; 6 Department of Paediatrics, Usmanu Danfodiyo University Teaching Hospital, Sokoto, Sokoto State, Nigeria; 7 Department of Paediatrics, University of Uyo and University of Uyo Teaching Hospital, Uyo, Akwa Ibom State, Nigeria; 8 Department of Paediatrics, Bowen University Teaching Hospital, Ogbomoso, Oyo State, Nigeria; 9 Department of Paediatrics, Rivers State University Teaching Hospital, Port Harcourt, Rivers state, Nigeria; 10 Department of Paediatrics, Abubakar Tafawa Balewa University, Bauchi State, Nigeria; 11 Department of Paediatrics, Federal Medical Centre Owo, Owo, Ondo State, Nigeria; 12 Department of Epidemiology & Community Health, University of Ilorin and University of Ilorin Teaching Hospital, Ilorin, Kwara, Nigeria; University of Illinois at Chicago, UNITED STATES

## Abstract

**Background:**

Despite the huge burden of sickle cell disease (SCD) among Nigerian children, the burden and outcome of respiratory illnesses remain undocumented. Thus, we aimed to describe the spectrum and outcome of respiratory illnesses among SCD childrenand adolescentadmissions in ten Nigerian tertiary hospitals.

**Method:**

A retrospective review of the SCD admission records of children and adolescents with a confirmed diagnosis of respiratory illnesses from 2012 to 2021 in ten tertiary health facilities across five geopolitical zones in Nigeria was conducted. The data, collectedbetween March and June 2023, included the age, sex, diagnosis, complications, duration and outcome of hospitalization.

**Results:**

Of the 72,333 paediatric admissions, 7,256 (10.0%) had SCD; the proportion of SCD from the total admission ranged from 2.1 to 16.3% in the facilities. Of the 7,256 children and adolescents with SCD, 1,213 (16.7%) had respiratory morbidities. Lower respiratory disease was the most common (70.0%) respiratory entity and the majority were pneumonia (40.1.0%), followed by acute chest syndrome (26.7%). Seventeen (1.4%) patients died; all had lower respiratory diseases [(acute chest syndrome ACS (11, 64.7%), pneumonia; 5, 29.4%, and asthma (1, 5.9%). Based on the proportion of deaths among overall SCD, the 17 death cases contributed 9.4% (95% CI 5.9 to 14.5). Factors associated with deaths included duration of hospitalization less than 72 hours and lower respiratory tract diseases.

**Conclusion:**

Sickle cell disease is a major contributor to hospitalization among Nigerian children and adolescents, with high respiratory morbidity and mortality. Pneumonia and acute chest syndrome were associated with mortality, andthe highest risk of death within the first 72 hours.

## Introduction

Sickle cell disease (SCD) is one of the most prevalent genetic diseases affecting children in sub-Saharan Africa [1]. Sickle cell disease is the most common inherited disorder of the blood, with an estimated 300,000 children born with the condition annually globally, of whom 70% occur in sub-Saharan Africa [2, 3]. Indeed, it is the most common genetic disorder affecting Nigerian children and occurs in 2.7–5.0% of the Nigerian population [4, 5].

The clinical manifestations of the disease arise from the tendency of sickled hemoglobin (HbS) to polymerize and deform red cells into the characteristic sickle shape [6]. The predominant clinical characteristic is vaso-occlusion (resulting in a painful crisis), hemolytic anaemia, and chronic inflammation [5–7]. Indeed, the disorder has multi-organ involvement, with the lungs being a frequent and serious organ affected [8].

The pulmonary complications of sickle cell disease result in significant morbidity and mortality in children [7–9]. Respiratory complications of sickle cell disease involve parenchymal, pulmonary vascular, and airway alterations with a progressive decline of lung function that often begins in childhood [8, 10]. Furthermore, respiratory infections such as pneumonia are a major cause of morbidity and mortality related to splenic dysfunction, impaired opsonization, sluggish alternative complement pathway activation, and lower serum immunoglobulin M (IgM) levels in SCD patients [8]. The commonly identified respiratory morbidities include acute chest syndrome (ACS), pneumonia, asthma/airway hyper-responsiveness, sleep-disordered breathing and pulmonary hypertension [8, 10].

The burden of respiratory problems in sickle cell disease is yet to be adequately established in Nigeria, despite SCD being a major genetic disease of the population. Sickle cell disease was the commonest co-morbid illness identified in some Nigerian studies on reviews of respiratory morbidities among emergency pediatric admissions, accounting for 3.7%-38.6% of the co-morbid illnesses [11, 12]. In Ife, Nigeria, acute chest syndrome (ACS) accounted for 11.3% of emergency admissions amongst SCD children, and 5.4% had asthma [7]. A study in Lagos, Nigeria, reported a 22.9% prevalence of pulmonary hypertension in children with sickle cell disease compared to 2.3% in non-SCD children [13].

Strategies for the appropriate care in the multidisciplinary approach to a chronic disorder such as sickle cell disease care require that clinicians are aware of the typical complications to anticipate. Early identification of children with sickle cell disease who have respiratory problems is crucial to enable prompt management to relieve hypoxia and treat crises. Hence, there is a need to identify the pattern of acute respiratory diseases among children with sickle cell disease. This is to enable the deployment of the necessary intervention needed for reducing the morbidity and mortality of respiratory illnesses, as timely and appropriate treatment is a key intervention. Thus, the objectives of this study were to identify the burden, spectrum and outcome of acute respiratory complications amongst children with sickle cell disease admitted in some tertiary hospitals across Nigeria.

## Materials and methods

### Study design and site

This was a descriptive cross-sectional study with a retrospective data collection over a maximum period of ten years (January 2012 to December 2021) and a minimum of four years (January 2018 to December 2021). This study was conducted in ten tertiary hospitals, randomly chosen from five regions of the country.

The country consists of 36 states located in six regions (North West, North East, North Central, South East, South West, and South-South) and Abuja, the capital of the country. Estimates of the population of Nigeria in 2022 were 225,082,083, with 53.5% living in urban areas and 46.5% in rural areas [14].

#### Inclusion criteria

Children with sickle cell disease hospitalized with a respiratory disease. For this study, we defined respiratory diseases as any condition affecting the respiratory tract, irrespective of etiology.

#### Exclusion criteria

Children with SCD managed as outpatients or those without hematologic confirmation of sickle cell disease.

### Method of data extraction

The records of all children and adolescents aged between 0 and 18 years admitted into the Emergency Paediatric Unit (EPU) with a diagnosis of sickle cell disease, irrespective of duration of stay, between 1st January 2012 and 31st December 2021 were retrieved from the admission registers of the respective hospital records. However, due to the commencement of electronic medical records in some centers and the loss of records, only two facilities had complete records for ten years.

The admission register contains the hospital number, name, gender, address, date of admission, diagnosis, outcome of hospital stay, and date of discharge of all hospitalized children. Each register is updated daily by the nurses and doctors. We also extracted the final diagnosis which contained the details of the child as a sickle cell disease patient with the associated morbidity and complications (S1 File). The final diagnosis was the diagnosis recorded after a review of the investigations by the managing team. The duration of stay for all the patients was obtained from the records. The total admissions of each year and the overall admissions of children and adolescents with sickle cell disease were also recorded (S2 File).

The diagnosis of respiratory diseases was based on a combination of clinical evaluation and relevant investigations, including but not limited to chest radiographs (repeated if acute chest syndrome suspected), chest computerized tomographic scans, Mantoux test, blood/ sputum cultures, Genexpert tests, spirometry, and echocardiograms, where indicated. The data extracted from the records were the age, sex, diagnosis, complications and co-morbidities, duration of stay, and outcome.A respiratory diagnosis with another respiratory complication in a subject was recorded as such in the proforma but analysed as two respiratory morbidities.The respiratory morbidities were classified as upper respiratory disease (consisting of acute upper respiratory tract infection and upper obstructive airway disorder) or lower respiratory disease (consisting of lower respiratory tract infections, and lower obstructive airway disorder).Also, the type of crises if present, was recorded; andthe crises were categorized as either vaso-occlusive or anaemic (sequestration, hyper-hemolytic, aplastic) type of crises [15]. The outcomes were categorized into three; discharged, died, and discharged against medical advice (DAMA).

Data collection occurred betweenthe 1^st^ of March and the 30^th^of June 2023.

### Data analysis

The data recorded on the proforma was entered into a microcomputer using numerical codes and analyzed with IBM SPSS version 23. The type of respiratory diseases, number of deaths, hospital discharges, and DAMA were expressed as frequencies and percentages. Continuous variables such as the age of the subjects and the duration of hospital stay were expressed as mean (Standard deviation) or median, interquartile range (IQR) depending upon whether the data was normally distributed or not, respectively. A chi-square test was used to check for an association between the demography and outcome parameters. A p-value of less than 0.05 was considered significant.

### Ethical considerations

Ethical approval was obtained from the Health Research Ethical Review Committee of the University of Ilorin Teaching Hospital ERC PAN/2023/02/0356.The ethics committee waived the requirement for informed consentas this was a retrospective review of medical records. The anonymity of the study participants was maintained.

## Results

### Distribution of SCD cases with respiratory presentations across the geopolitical zones

The study included 1,213 children with sickle cell disease seen in the emergency pediatric units in 10 tertiary facilities over a maximum ten-year interval (2012–2021). Two facilities provided data for 10 years, and five facilities provided data for 6 years (Table 1). The largest contributions to the number of subjects recruited were from Kwara (32.4%), Gombe (18.5%), Sokoto (15.6%), and Katsina (15.3%), collectively accounting for 81.8% of subjects.

**Table 1:** Distribution of cases across the recruiting states.

States	Period covered	Duration (years)	Number of subjects (percentage contribution)
Bauchi	2018 to 2021	4	25 (2.1)
Gombe	2016 to 2021	6	224 (18.5)
Sokoto	2012 to 2021	10	189 (15.6)
Katsina	2015 to 2021	7	186 (15.3)
Niger	2016 to 2021	6	40 (3.3)
Kwara	2012 to 2021	10	393 (32.4)
Rivers	2018 to 2021	4	84 (6.9)
Akwa Ibom	2016 to 2021	6	32 (2.6)
Oyo	2016 to 2021	6	23(1.9)
Ondo	2016 to 2021	6	17 (1.4)
**Total**			**1,213 (100.0)**

### Proportion of admissions with sickle cell disease and respiratory morbidity

There were 72,333 admissions of children into the Emergency Paediatric Units of the study facilities over the period. The percentage of these admissions that had sickle cell disease was 10.0% (7,256 patients) and ranged from a low of 2.1% in Niger State to a high of 16.3% in Kwara State (Table 2). Children with sickle cell disease and respiratory complications constituted 16.7% (1,213 subjects) of the 7,256 children with sickle cell disease admitted. This ranged from the lowest prevalence of 6.8% in Sokoto State to the highest recorded prevalence of 68.9% in Rivers State. The distribution of morbidity is shown in Table 2.

**Table 2:** Proportion of admissions with sickle cell disease and respiratory morbidity.

States	Number of admissions	Admissions with SCD	Percentage of overall admissions with SCD	Admissionswith SCD and respiratory complications	Percentage of admissionswith SCD that had respiratory complications
Bauchi	3,964	269	6.8%	25	9.3%
Gombe	6,785	602	8.9%	224	37.2%
Sokoto	16,124	2,430	15.1%	189	7.8%
Katsina	6,879	857	12.5%	186	21.7%
Niger	9,396	198	2.1%	40	20.2%
Kwara	13,361	2,182	16.3%	393	18.0%
Rivers	1,381	122	8.8%	84	68.9%
Akwa Ibom	4,539	228	5.0%	32	14.0%
Oyo	4686	240	5.1%	23	9.6%
Ondo	5218	128	2.5%	17	13.3%
**Overall**	**72,333**	**7,256**	**10.0%**	**1213**	**16.7%**

### General characteristics of the children with SCD and respiratory morbidity

The median age was 70.0 months with IQR (36.0–120.0). Most of the subjects were five years and above (705, 58.1%). Males (56.3%) were slightly more than females. Lower Respiratory disease constituted more than two-thirds of emergency presentations among the children with sickle cell disease (Table 3**)**. Seventeen (1.4%) patients died, 98.4% (1,194) were discharged after full recovery, and two (0.4%) were discharged against medical advice (DAMA).

**Table 3:** Demographic characteristics and outcome of the SCD-associated respiratory morbidity in the subjects.

Variable	Frequency (%)	Died N = 17	Survived N = 1196	OR (95%CI)	p-value
**Age group (years)**					
< 5	508 (41.9)	4 (23.5)	504(42.5)	0.42 (0.14–1.30)	0.122
≥ 5	705 (58.1)	13 (76.5)	692 (57.5)		
**Sex**					
Male	683 (56.3)	10 (58.8)	673 (56.3)	1.11 (0.42–2.94)	0.833
Female	530 (43.7)	7 (41.2)	523(43.7)		
**Respiratory Disease Categories**					
Upper Respiratory	361 (29.8)	0 (0.0)	361 (30.2)	0.14 (0.01–0.78)	**0.005**
Lower Respiratory	852 (70.2)	17 (100.0)	835 (69.8)		
**Crises present**	267 (22.0)	3 (17.6)	264(22.1)	0.76 (0.22–2.65)	0.931
* **Co-morbid illness**	169 (13.9)	1 (5.9)	168 (14.0)	0.38 (0.05–2.90)	0.581
**Duration of hospital stay (days)**					
<3	172(14.2)	12 (70.6)	160(13.4)	15.54 (5.40–44.70)	**<0.001**
≥3	1041(85.8)	5(29.4)	1036 (86.6)		

*Co-morbid illnesses were **infectious** (161):malaria = 91, diarrhea = 24, urinary tract infection = 17, osteomyelitis-14, measles = 5, septic arthritis = 2, HBV = 2, meningitis = 2, cellulitis = 1, typhoid enteritis = 1, myocarditis = 1, Pott’s disease = 1 and **non-infectious** (8):Cerebrovascular accident = 4,congenital diaphragmatic hernia = 1, ventricular septal defect = 1, ricket = 1, Down syndrome = 1

The median duration of hospital stay was 6.0 (3.0–9.0) days. The children that died were in the age groups less than five years (four, 23.5%), 5–10 years age group (six, 35.7%), and seven (41.2%) children aged 10 years and above. All deaths occurred within the first four days of admission and were exclusively lower respiratory diseases. There was a significant relationship between the respiratory disease category (upper and lower respiratory disease) and outcome, p **= 0.004**, respectively. Age categories, gender, the presence of co-morbidity/complication and crisis had no relationship with death, p>0.05.

#### Crises in SCD

**Fig 1:**
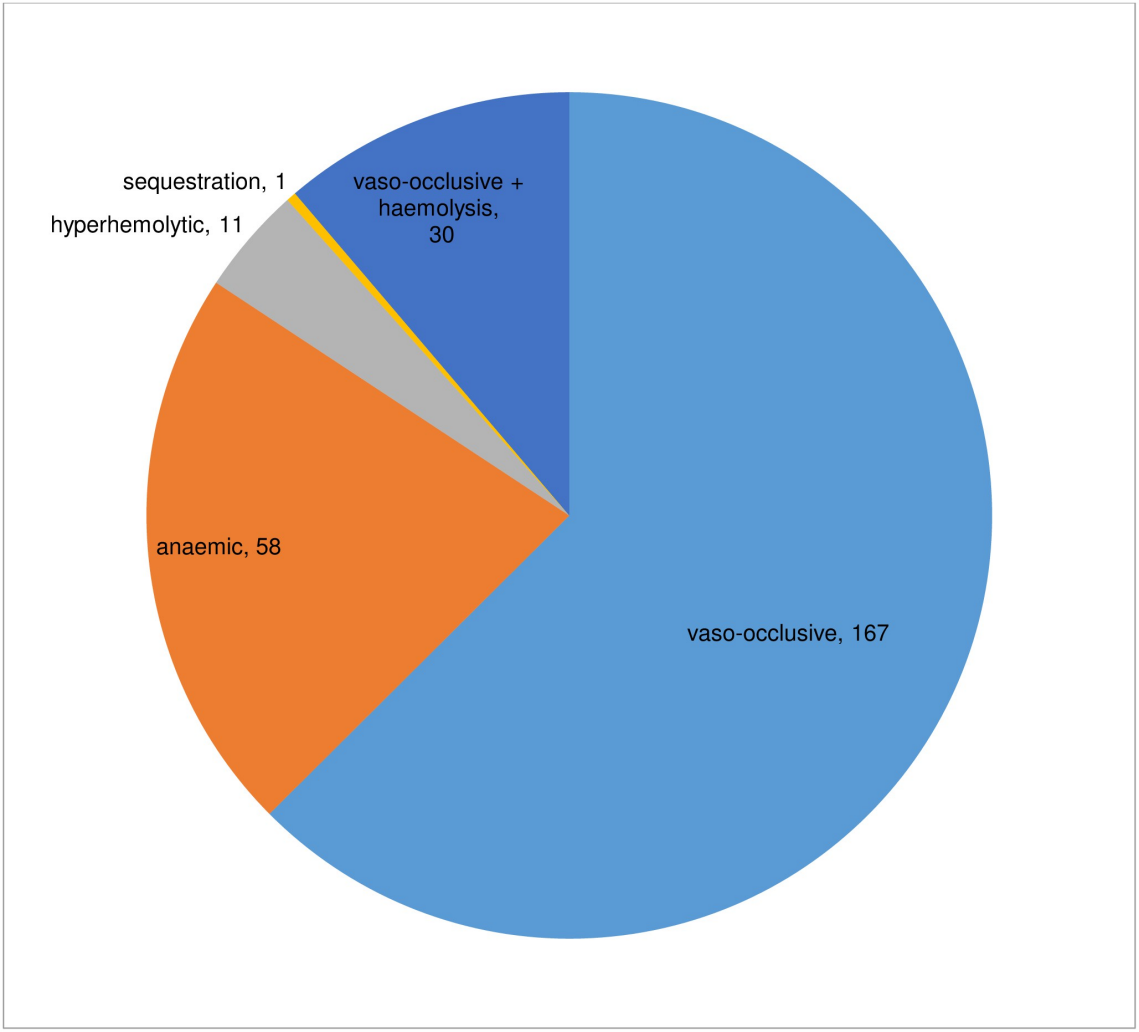
Pie chart showing the frequency of crisis in those with respiratory morbidity.

Among the 1213 children with SCD and respiratory morbidity, 267(22.0%) had a crisis. The most common type was the vaso-occlusive crisis identified in 167 (62.5%) children while the least common was sequestration (1, 0.4%), “Fig 1”. Three of the 17 who died had a crisis, each of VOC, anaemic, and mixed (VOC +anaemic).

### Spectrum of respiratory disease among the study population

A total of 1251 respiratory morbidities were noted among the 1213 children; a single respiratory morbidity occurred in 1176 (96.9%) children and 37 (3.1%) children had two respiratory morbidities. Lower respiratory disease was the most common (70.0%) respiratory entity identified among the study population. Among the lower respiratory diseases, the majority were pneumonia (40.1%), followed by acute chest syndrome (26.7%). Acute pharyngitis/tonsillitis was the most common upper respiratory disease among the study population, and the others are shown in Table 4. All mortality occurred in patients with lower respiratory diseases, with the leading causes being acute chest syndrome (ACS) (11, 64.7% of 17 deaths) and pneumonia (five subjects; 29.4%).

**Table 4:** Spectrum of respiratory disease among the children with SCD.

Type of respiratory morbidity		Frequency	Deaths
**Upper Respiratory Disease**		**375 (30.0)**	**0 (0.0)**
Infective	Acute pharyngitis/tonsillitis	297(23.7)	0(0.0)
	URTI (Unclassified)	54 (4.3)	0 (0.0)
	Acute otitis media	7 (0.6)	0 (0.0)
	Rhinitis	3 (0.2)	0 (0.0)
	Acute laryngotracheobronchitis	2 (0.2)	0 (0.0)
	Retropharyngeal abscess	1 (0.1)	0 (0.0)
	Peritonsillar abscess	1 (0.1)	0 (0.0)
	Mumps	1 (0.1)	0 (0.0)
	Pertussis	1 (0.1)	0 (0.0)
Non-infective	Epistaxis	4 (0.2)	0 (0.0)
	Adenoidal Hypertrophy	4(0.2)	0 (0.0)
**Lower Respiratory Disease**		**876 (70.0)**	**17 (100.0)**
Infective	Pneumonia	502(40.1)	5 (29.4)
	Pleural effusion	9 (0.7)	0 (0.0)
	Empyema	5 (0.4)	0 (0.0)
	Bronchiolitis	4 (0.3)	0 (0.0)
	Pulmonary tuberculosis	4 (0.3)	0 (0.0)
	Pneumothorax	1 (0.1)	0 (0.0)
Non-infective	Acute chest syndrome (ACS)	334 (26.7)	11 (64.7)
	Asthma	12 (1.0)	*1 (5.9)
	Aspiration Pneumonitis	3 (0.2)	0 (0.0)
	Pulmonary hypertension	2 (0.2)	(0.0)

*Patient had co-morbid tuberculosis of the spine.

### Distribution of overall mortality, SCD mortality, and SCD respiratory-related mortality

Overall, there were 7,045 deaths from all causes of the 72,333 admissions recorded in all the sites, representing a 9.7% (95% CI 9.5 to 10.0) mortality rate (Table 5). Excluding subjects with sickle cell disease, there were 65,077 admissions and 6,864 deaths (10.5%; 95% CI 10.3, 10.8). Amongst the subjects with sickle cell disease, there were 7,256 admissions and 181 deaths (2.5%, 95% CI 2.2, 2.9). Respiratory-related conditions were responsible for deaths in 17 subjects with sickle cell disease, contributing 9.4% (95%CI 5.9, 14.5) of the 181 deaths in subjects with SCD (Table 5). The facility with the highest respiratory contributor to deaths in children with SCD of 20.0% was in Katsina State (Table 5).

**Table 5:** Distribution of overall deaths, SCD-deaths, and SCD respiratory-related deaths according to facility.

Variable	Total admissions	Number of deaths (all admissions)	Percentage mortality (all admissions)	Number of deaths (SCD)	Percentage mortality (SCD)	Number of Respiratory-related deaths in SCD	Contribution of respiratory-related deaths in SCD to total deaths in SCD
Bauchi	3,964	465	11.7%	1	0.4%	0	0.0%
Gombe	6,785	1,403	20.7%	24	4.0%	3	12.5%
Sokoto	16,124	931	5.8%	75	3.1%	2	2.7%
Katsina	6,879	644	9.4%	25	2.9%	5	20.0%
Niger	9,396	1000	10.6%	3	1.5%	0	0.0%
Kwara	13,361	1,248	9.3%	38	1.7%	6	15.8%
Rivers	1,381	21	1.5%	2	1.6%	0	0.0%
Akwa Ibom	4,539	224	4.9%	6	2.6%	1	16.7%
Oyo	4,686	781	16.7%	3	1.3%	0	0.0%
Ondo	5,218	328	6.3%	4	3.1%	0	0.0%
**Total**	**72,333**	**7,045**	**10.0%**	**181**	**2.5%**	**17**	**9.4%**

The odds of mortality were significantly lower in the children with sickle cell disease compared to non-sickle cell disease admissions (OR 0.22; 95%CI 0.19–0.25) (Table 6). Also, the odds of mortality were reduced in SCD with respiratory complications as compared to those without respiratory complications (OR 0.51; 95%CI 0.31, 0.84).

**Table 6:** Contribution of SCD and respiratory complications of SCD to mortality.

Admissions	Died (%)	Alive (%)	OR(95% CI)	P
**All admissions**				
Subjects with SCD	181(2.5)	7075 (97.5)	0.22(0.19, 0.25)	**<0.001**
Subjects without SCD	6864 (10.5)	58213 (89.5)		
**Total**	**7045 (9.7)**	**65288 (91.3)**		
**SCD admissions**				
SCD with respiratory complication	17 (1.4)	1196(98.6)	0.51(0.31, 0.84)	**0.005**
SCD without a respiratory complication	164 (2.7)	5879 (97.8)		
**Total**	**181 (2.5%)**	**7075 (97.5)**		

## Discussion

Nigeria has a high global burden of SCD with attendant complications that may necessitate the need for in-patient care. From this multi-centre study, the average contribution of sickle cell disease to hospital admissions in this study is higher compared with a single-centre study in Iraq [16]. This finding shows that despite all efforts to reduce the complications associated with sickle cell disease, it continues to contribute to overall morbidity among Nigerian children and is probably a reflection of the high burden of sickle cell disease in the country [2, 5].

Respiratory complications in children with sickle cell disease are relatively high in this study, with a prevalence of 16.7% and a range of 6.8–68.9% across the centres studied. This finding is in tandem with the report of the United States of America (USA) sickle cell expert panel documenting that respiratory conditions were found in children with sickle cell disease with a prevalence of 20–48% and are associated with mortality [17]. The variation in respiratory morbidities is also similar to earlier report by Adesanya *et al* that ARI in under-five Nigerian children residing in North central region, North eastern region and Southern region were associated with having higher risk of ARI symptoms than children from the South western region [18]. This spatial variation in the North regions of Nigeria may be due to higher levels of dust exposure from the dust-laden north-east trade winds from the Sahara Desert, the predominantly dry Northern region, and sandstorms which could predispose to respiratory morbidities [19, 20]. A very high prevalence of respiratory morbidities (68.9%) was identified in one of the study centres (Rivers State, Nigeria), which may be attributable to the high air pollution levels prevalent in the region [21, 22]. Indeed, this current figure for respiratory morbidities compares with the prevalence of acute respiratory infections (67%) in children earlier reported in the same city by Abereton*et al* [23]; 74.5% households had visible soot marks. Short-term particulate matter (PM) exposure in children reportedly has increased respiratory health effects, including cough, respiratory hospital admissions, and wheeze compared with adults [24, 25]. The state (Rivers) is oil-producing, and the petroleum industry constitutes a major source of air pollution from production operations, such as oil and condensate spills, and gas flaring, and automobile transportation [22]. Moreso, the residents have experienced particle (soot) emissions since 2016 [26].

Different studies have shown that respiratory complications are one of the leading causes of hospital admission in children [16, 27]. The respiratory morbidity, either upper or lower respiratory tract infection, is often the admission diagnosis precipitating an episode of painful crisis as earlier reported [16, 27]. Lower respiratory tract infection constituted the major reason for admission in this study. Lower respiratory tract infections in sickle cell disease have been attributed to altered complement activation with reduced oxygen tension. The reduced oxygen tension leads to increased polymerization, tissue inflammation and pulmonary bed infarction [28]. This current study result is similar to the findings by Abd El-Ghany*et al*, [27] and Hawasawi*et al*, [29] who observed pneumonia as a leading cause of admission in children with sickle cell disease.

While lower respiratory tract infections are common in sickle cell disease, infections have also been implicated in the pathogenesis of acute chest syndrome in children with sickle cell disease [30]. Pneumonia often clinically resembles acute chest syndrome presenting with similar features [30]. However, acute chest syndrome often defined as “the presence of a new pulmonary infiltrate on chest X-ray, in addition to one or more of the following symptoms: chest pain, a temperature of more than 38.5◦C, tachypnea, wheezing, or cough” [30] tend to be more severe with multilobular affectation and a likely progression to respiratory failure [31]. Hence, pneumonia poses an ominous danger to the survival of children with sickle cell anaemia because the acute chest syndrome that it mimics is a known life-threatening acute pulmonary complication of sickle cell disease [30–32]. Thus, for every SCD admission with pneumonia, the clinician should monitor the child for worsening hypoxaemia, difficulty breathing and changes in the chest radiograph.

Acute chest syndrome was the second most common lower respiratory tract condition in this study. This finding is similar to those of Farooq *et al* [33] and Quinn *et al*, [34] who reported acute chest syndrome as the second commonest cause of admission, the commonest cause of admission into the intensive care unit and the commonest cause of mortality in children older than two years. Acute chest syndrome is a difficult-to-distinguish, pneumonia-like disease in sickle cell disease and frequently contributes to morbidity and hospitalization in children [27]. Unlike in adults, where infarction heralds acute chest syndrome, more than one-third of cases of acute chest syndrome in children are caused by viral, mycoplasma and chlamydia infection pneumonia [33]. Thus, prevention and universal prompt treatment of pneumonia is highly recommended in children with sickle cell disease with suspected acute chest syndrome as it contributes significantly to the mortality of children with sickle cell disease [33].

Bronchial asthma was the second most common non-infective respiratory cause of hospital admission in this study. Bronchial asthma is a significant co-morbidity in sickle cell disease children, occurring in more than one-third of children, and is an independent predictor of mortality [35, 36]. The high burden of acute chest syndrome and bronchial asthma in this study may be due to the positive bidirectional relationship between the two morbidities in children with sickle cell disease. Duckworth *et al* have shown that of the children with sickle cell disease and physician-diagnosed bronchial asthma, more than four-fifths (85%) had acute chest syndrome [35]. This bidirectional relationship has been linked to ventilation-perfusion mismatch in asthma, causing increased sickling of red cells leading to acute chest syndrome, and increased inflammatory mediators in acute chest syndrome exacerbating airway hyperreactive airways characteristic of bronchial asthma [37].

Pharyngitis and tonsilitis were the most common upper respiratory tract infections in this study. This may be because pharyngitis and tonsilitis are generally viral in etiology in children, viral diseases are commoner in children and the developing countries where this study was conducted account for 90% of the global burden [38]. This also explains why upper respiratory infections which are primarily bacterial in origin, such as retropharyngeal and parapharyngeal abscesses, were less frequent in this study. The presence of varied bacterial upper respiratory tract infections in this study may be a pointer to the defective alternate complement pathways in sickle cell disease children and associated immunocompromised states. The finding in the current study is similar to the report by Alkindi *et al*. [28].

The percentage contribution of sickle cell mortality to the overall childhood mortality was 2.5% in this study. This finding is lower compared to the report by Obiageli *et al*, in a model-estimated and population-analysis of data, of 4.2% in Nigeria, and 5–10% in many sub-Saharan African countries [39]. This disparity might be because Obiageli *et al* reported the under-five mortality attributable to excess mortality from sickle cell disease, unlike the current study that documented the sickle cell disease mortality rate in all children below the age of 18 years, making the finding more representative of the percentage contribution of sickle cell to overall childhood mortality. The higher under-five mortality attributable to sickle cell disease by Obiageli *et al* might be because mortality in children with sickle cell disease has been reported to be inversely related to age [40]. Indeed, the odds of mortality were reduced in SCD with respiratory complications as compared to those without respiratory complications, as well as SCD compared with the non-SCD in the current study. A plausible reason for this finding in children with SCD and respiratory morbidity may be due to the possibility of early presentation among the SCD cohort, which is often advocated as part of the health education to their parents at each clinic. Others are the aggressive use of broad-spectrum antibiotics, or their being on penicillin V prophylaxis which are advocated for compared to the non-SCD group. However, this should be interpreted with caution as the retrospective design of the study precludes an analysis of confounders.

Respiratory-related mortality, and exclusively lower respiratory condition, is the leading cause of mortality in sickle cell disease in this study. This finding is not surprising as the commonest cause of general admission and the commonest cause of admission into the intensive care unit among sickle cell disease children has been attributed to acute chest syndrome, which is a lower respiratory condition [33, 34]. Other studies have reported pulmonary infection to be the leading cause of mortality because the most frequent portal of entry in children is the respiratory tract [40, 41]. The mortality recorded in this study occurred within the first three days of admission. This finding is similar to the findings of Manci *et al* in the USA of early death after admission as a common occurrence in sickle cell disease necessitating a high index of suspicion in sickle cell disease children in the first few days of admission [41].

## Conclusion

This study demonstrated there exists a high burden of SCD among paediatric emergency hospital admissions in Nigeria with high respiratory morbidity but relatively low associated death. Pneumonia and acute chest syndrome were the two main respiratory conditions associated with mortality and the highest risk of death occurring within the first 72 hours.

### Strengths of the study

This study was multi-centred, spanning five of the six geopolitical zones, and therefore representative of the burden of respiratory diseases among children with SCD in Nigeria.

### Limitations of the study

This was a retrospective study, which depends on the accuracy of written data and some important data might have been lost in the process of storage. We tried to overcome this limitation by ensuring that data were retrieved from both electronic medical and manual health record management systems in the facilities involved.

## Supporting information

S1 File The data of the SCD children from the ten facilities. (XLSX)

S2 File The data on overall admissions and deaths in the ten facilities. (XLSX)
